# Peptide Receptor Radionuclide Therapy Targeting the Somatostatin Receptor: Basic Principles, Clinical Applications and Optimization Strategies

**DOI:** 10.3390/cancers14010129

**Published:** 2021-12-28

**Authors:** Niloefar Ahmadi Bidakhvidi, Karolien Goffin, Jeroen Dekervel, Kristof Baete, Kristiaan Nackaerts, Paul Clement, Eric Van Cutsem, Chris Verslype, Christophe M. Deroose

**Affiliations:** 1 Department of Nuclear Medicine, University Hospitals Leuven, 3000 Leuven, Belgium; niloefar.ahmadibidakhvidi@uzleuven.be (N.A.B.); karolien.goffin@uzleuven.be (K.G.); kristof.baete@uzleuven.be (K.B.); 2 Nuclear Medicine and Molecular Imaging, Department of Imaging and Pathology, KU Leuven, 3000 Leuven, Belgium; 3 Department of Digestive Oncology, University Hospitals Leuven, 3000 Leuven, Belgium; jeroen.dekervel@uzleuven.be (J.D.); eric.vancutsem@uzleuven.be (E.V.C.); chris.verslype@uzleuven.be (C.V.); 4 Department of Respiratory Oncology, University Hospitals Leuven, 3000 Leuven, Belgium; kristiaan.nackaerts@uzleuven.be; 5 Department of General Medical Oncology, University Hospitals Leuven, 3000 Leuven, Belgium; paul.clement@uzleuven.be

**Keywords:** PPRT, peptide receptor radionuclide therapy, ^177^Lu-DOTATATE, ^90^Y-DOTATOC, NET, neuroendocrine tumor, NETTER-1, peptide receptor chemoradionuclide therapy

## Abstract

**Simple Summary:**

Peptide receptor radionuclide therapy (PRRT) is a systemic treatment consisting of the administration of a tumor-targeting radiopharmaceutical into the circulation of a patient. The radiopharmaceutical will bind to a specific peptide receptor leading to tumor-specific binding and retention. This will subsequently cause lethal DNA damage to the tumor cell. The only target that is currently used in widespread clinical practice is the somatostatin receptor, which is overexpressed on a range of tumor cells, including neuroendocrine tumors and neural-crest derived tumors. Academia played an important role in the development of PRRT, which has led to heterogeneous literature over the last two decades, as no standard radiopharmaceutical or regimen has been available for a long time. This review focuses on the basic principles and clinical applications of PRRT, and discusses several PRRT-optimization strategies.

**Abstract:**

Peptide receptor radionuclide therapy (PRRT) consists of the administration of a tumor-targeting radiopharmaceutical into the circulation of a patient. The radiopharmaceutical will bind to a specific peptide receptor leading to tumor-specific binding and retention. The only target that is currently used in clinical practice is the somatostatin receptor (SSTR), which is overexpressed on a range of tumor cells, including neuroendocrine tumors and neural-crest derived tumors. Academia played an important role in the development of PRRT, which has led to heterogeneous literature over the last two decades, as no standard radiopharmaceutical or regimen has been available for a long time. This review provides a summary of the treatment efficacy (e.g., response rates and symptom-relief), impact on patient outcome and toxicity profile of PRRT performed with different generations of SSTR-targeting radiopharmaceuticals, including the landmark randomized-controlled trial NETTER-1. In addition, multiple optimization strategies for PRRT are discussed, i.e., the dose–effect concept, dosimetry, combination therapies (i.e., tandem/duo PRRT, chemoPRRT, targeted molecular therapy, somatostatin analogues and radiosensitizers), new radiopharmaceuticals (i.e., SSTR-antagonists, Evans-blue containing vector molecules and alpha-emitters), administration route (intra-arterial versus intravenous) and response prediction via molecular testing or imaging. The evolution and continuous refinement of PRRT resulted in many lessons for the future development of radionuclide therapy aimed at other targets and tumor types.

## 1. Peptide Receptor Radionuclide Therapy: Concept and Early Development

Peptide receptor radionuclide therapy (PRRT) is a systemic treatment consisting of the administration of a tumor-targeting radiopharmaceutical into the circulation of a patient. The radiopharmaceutical will bind to a specific peptide receptor leading to tumor-specific binding and retention. Examples of receptors that have been studied include the somatostatin receptor (SSTR), glucagon-like peptide-1 receptor (GLP-1R), bombesin receptor, cholecystokinin type 2 (CCK2R) and melanocortin receptor [1]. The only target that is currently being used in clinical practice is the SSTR, and this review will focus on PRRT for this target.

The SSTR is overexpressed in neuroendocrine tumors (NETs), which arise from neuro-endocrine cells that are present in a range of organs, including the gastro-intestinal tract and pancreas (GEP-NETs, both functional and non-functional), the bronchi (typical and atypical carcinoid tumors, large cell neuroendocrine carcinoma and small cell lung cancer), NET of unknown primary tumor (CUP-NET) and arising from more less frequent primary sites (thymus, breast, etc.) [2,3]. There are five subtypes of SSTRs in humans [4], the most important subtype for theranostics applications being subtype 2 (Table 1). Other tumoral entities with high SSTR-overexpression include neural-crest derived tumors (phaeochromocytoma, paraganglioma, neuroblastoma), meningioma, medullary thyroid cancer, Merkel cell carcinoma, with anecdotal evidence of overexpression in other tumor types (renal cell carcinoma, gastro-intestinal stromal tumors).

The overexpression of the SSTR in GEP-NETs has been used in different manners. Treatment of patients with metastatic disease using non-radioactive somatostatin analogues (SSAs) results in control of symptoms induced by hormonal release by these tumors and leads to an anti-proliferative effect in non-functional tumors [8,9]. In the late 1980s and early 1990s, the potential for exploiting the SSTR for radionuclide-based applications was put forward by the Rotterdam group. Diagnostic in vivo imaging was first reported using ^123^I-Tyr^3^-octreotide gamma-camera scintigraphy in meningiomas and GEP-NETs, followed by more extensive data in small intestine NET (SI-NET), pancreatic NET and paragangliomas [10,11]. ^111^In-pentetreotide was rapidly developed as alternative imaging agent with a longer half-life, showing similar clinical value [12]. The current state-of-the art diagnostic imaging of the SSTR is done with positron emission tomography (PET) ligands, SSAs labeled with either gallium-68 or other positron emitters (copper-64, fluorine-18, etc.) [13,14,15].

Demonstrable high uptake and retention of radiolabeled SSAs in tumoral tissue and only limited uptake in normal organs, mainly endocrine organs such as pituitary gland, adrenals, pancreas and thyroid gland, opened an attractive avenue, i.e., the treatment of patients with SSTR-positive tumors as demonstrated by imaging with subsequent radionuclide therapy (RNT) [16]. Initial results with the Auger emitter ^111^In-pentetreotide were promising with beneficial effects on clinical symptoms, hormone production [16] and tumor size, including size reduction in 6 out of 21 patients treated with sufficiently high injected activity (29%) [17]. Due to the very limited penetration range in tissue of Auger electrons (nanometer to short micrometer), Auger-emitters should be located within the subcellular compartment of the target region (e.g., nucleus for DNA irradiation) [18]. Since agonist SSAs however lead to internalization into the cytoplasm, but not the nucleus, it was rapidly realized that beta-emitting radiopharmaceuticals, with longer ranges, might generate better clinical results. This led to the development of second generation ^90^Y-DOTA-Tyr^3^-Octreotide (^90^Y-DOTATOC) [19], using the high energy pure β^-^emitter yttrium-90 (^90^Y; E_max_: 2.28 MeV; E_mean_: 0.935 MeV) with tissue penetration of ~11 mm [20]. Initial patient results in a phase-I trial (*n* = 20) showed good results with partial response (PR) in 5 patients (25%) and stable disease (SD) in 11 patients (55%), with limited toxicity [21]. However, the high accumulation of radiolabeled SSAs in the kidney, combined with the long range of electrons from yttrium-90 and the lack of γ-emission to perform patient-based dosimetry, raised concerns for potential radiation-induced nephrotoxicity. Initial reports of end-stage renal disease (ESRD) were confirmed by the data from the Basel group in a series of 1109 patients, in which 102 (9.2%) developed ESRD [22,23]. Thus, the third generation ^177^Lu-DOTA-Tyr^3^-octreotate (^177^Lu-DOTATATE) was seen as an attractive alternative, due to improved affinity for SSTR and more importantly, the lower energy of the electrons from lutetium-177 (^177^Lu; multiple β emissions with E_max_: 0.497 Mev and E_mean_: 0.133 Mev;) and the resulting lower tissue penetration [20,24]. Furthermore, decay of lutetium-177 is associated with γ-emission (113 and 208 keV at 6.4% and 11% yield, respectively) [20] and thus allows dosimetry of actual absorbed dose of tumors and organs at risk, such as the kidneys, on a patient-specific basis. This radiopharmaceutical has emerged as the current clinical standard and is the only one authorized by the American Food and Drug Administration (FDA) and European Medicines Agency (EMA).

α-emitters are the most potent category of radionuclides for RNT. In an attempt to increase the potential of PRRT, recently α-emitting SSAs have been explored in small patient cohorts. This fourth generation of PRRT radiopharmaceuticals includes ^213^Bi-DOTATOC, ^225^Ac-DOTATATE/-TOC and ^212^Pb-DOTAMTATE [25,26,27,28]. Although there is only very limited patient data at present, these α-emitting SSAs are very promising as they can overcome resistance to β-emitters and might have higher objective response rates (ORRs) than β-emitters [25,26,28].

**Figure 1:**
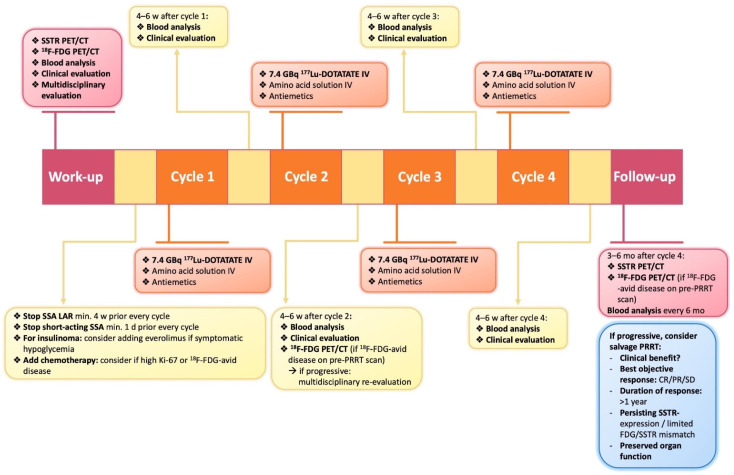
Proposed standardized PRRT scheme. IV = intravenous; SSA = somatostatin analogue; LAR = long-acting release; mo = months; w = weeks; d = day; PRRT = peptide receptor radionuclide therapy; CR = complete response; PR = partial response; SD = stable disease; SSTR = somatostatin receptor; FDG = fluorodeoxyglucose.

The development of PRRT has seen a large involvement of academic centers, both for the development of the radiopharmaceuticals and the clinical trials. This academia-driven development, in contrast to the development of most novel anti-cancer drugs that have been industry-driven, has led to heterogeneous literature as no standard radiopharmaceutical or regimen has been available for a long time (Figure 1). The heterogeneity in the literature is also caused by prolonged phase-II trials involving PRRT and by the interference of logistic barriers (e.g., access to radiopharmaceutical and reimbursement) in the clinical use of PRRT. The development of PRRT and its continuous refinement has been of great clinical value for patients treated with PRRT. However, it is also a paradigm-shaping therapeutic strategy, where theranostic pairs are used for image-based patient selection and subsequent RNT. This concept can be extended to other targets and tumor types, as has been recently demonstrated by ^177^Lu-PSMA-617 treatment of prostate-specific membrane antigen (PSMA)-expressing prostate carcinoma patients [29,30].

This review focuses primarily on the clinical studies involving PRRT for the somatostatin receptor. In addition, multiple optimization strategies for PRRT are discussed and interesting preclinical avenues are introduced where appropriate.

## 2. Current Evidence

### 2.1. Indications

Patients with advanced NET and clinically, biochemically, or radiographically progressive disease after first line treatment with SSAs, are eligible for second line treatment with PRRT if sufficient uptake on somatostatin receptor scintigraphy (SRS) is present [31]. Eligibility for PRRT is determined via mandatory pre-treatment SSTR imaging, preferentially by SSTR PET, blood analysis and clinical evaluation. ^18^F-fluorodeoxyglucose (^18^F-FDG) PET/CT provides additional information, and all lesions should show sufficient SSTR expression, in particular the ^18^F-FDG-avid ones. In addition, the concept of neo-adjuvant and pseudo-neoadjuvant treatment with PRRT in patients with initially (borderline) operable and inoperable tumors, respectively, has been investigated in a limited number of small patient cohorts [32,33,34]. Van Vliet et al. retrospectively investigated pseudo-neoadjuvant treatment with PRRT in 29 patients with nonfunctioning pancreatic NETs, leading to successful surgery in 31% (9/29) of the patients and a median progression-free survival (PFS) of 69 months compared to 49 months in the other patients [32]. The median length between the last cycle of PRRT and surgery was 12 months (range 7–33 months). Additional prospective studies are needed to further investigate the use of PRRT as a tool to render inoperable tumors amenable to surgery.

### 2.2. Efficacy and Outcome

#### 2.2.1. Response Assessment

Low grade NETs are known to be slowly growing, hereby often exhibiting a delayed response on morphological imaging after treatment. Currently, response assessment via morphological and/or molecular PET imaging is typically performed one to three months after completion of PRRT [35]. Historically, a morphological decrease in tumor size is commonly associated with an increase in survival, ultimately reflecting treatment efficacy. At present, the Response Evaluation Criteria in Solid Tumors (RECIST) methodology is still considered the gold standard for response assessment in solid tumors [36]. However, several PRRT studies have used the Southwest Oncology Group (SWOG) criteria for response assessment in NETs. The one-dimensional (RECIST) versus two-dimensional (SWOG) measurement of lesions is the main difference between these two response criteria. Van Vliet et al. compared four different response criteria (RECIST, SWOG, mRECIST (modified RECIST) and mSWOG (modified SWOG)) in patients with NETs treated with ^177^Lu-DOTATATE [37]. The different response criteria revealed similar results and predicted PFS and overall survival (OS) in a comparable manner. Notably, PFS and OS were comparable in patients with objective response and SD. Nonetheless, response assessment after PRRT solely based on tumor shrinkage may not be sufficient, given the fact that changes in tumor perfusion and tumor necrosis are not considered [38]. Furthermore, the nadir of the tumor shrinkage occurs late after treatment, from several months to even several years [39]. Response criteria for PET imaging of NETs are highly desirable. The difficulty with molecular imaging of the SSTR is based on the fact that a quantitative decrease in ligand uptake on PET imaging after PRRT can be caused by a therapeutic effect but also by disease progression, perfusion changes or dedifferentiation [38]. SSTR PET imaging is useful to document progression through the appearance of new lesions. However, currently no significant correlation has been found between changes in SSTR uptake on PET after PRRT and patient outcome [40,41]. Further studies are awaited to determine the role of PET imaging in response assessment after PRRT, also the role of radiomics needs to be explored in an extensive manner in this setting. At present, the role of circulating biomarkers (Chromogranin A (CgA), Neuron Specific Enolase (NSE), 5-Hydroxyindole-3-Acetic Acid (5-HIAA)) in response assessment after PRRT is limited, due to low sensitivity and specificity [42]. New techniques involving liquid biopsies are being studied [38].

#### 2.2.2. ^90^Y-DOTATOC

The Basel group performed a phase-II single-center open-label trial with ^90^Y-DOTATOC in a large cohort of 1109 patients with metastasized neuroendocrine cancers [23]. A median of 2 cycles were administered per patient (range 1–10), with an activity of 3.7 GBq/m^2^/cycle ^90^Y-DOTATOC (Table 2). Morphological response was observed in 34.1%, biochemical response in 15.5% and clinical response in 29.7% of the patients. Morphological, biochemical and clinical response were significantly correlated with a longer survival. Tumor and kidney uptake on baseline SRS (Octreoscan^®^) were predictors for survival and nephrotoxicity, respectively [23]. Further, a post hoc analysis of a prospective phase-II trial was performed in our center including 43 patients treated with ^90^Y-DOTATOC [43]. Patients received up to four cycles of ^90^Y-DOTATOC at 1.85 GBq/m^2^/cycle with a kidney biologically effective dose (BED) of maximum 37 Gy. A disease control rate (DCR) of 55% was observed. High ^68^Ga-DOTATOC tumor uptake on baseline imaging was independently associated with a better survival after treatment with ^90^Y-DOTATOC. Median PFS and OS were 13.9 months and 22.3 months, respectively [43]. Overall ORRs between 4–34% for ^90^Y-DOTATOC have been reported in literature [23,44]. However, head-to-head comparisons between studies, specifically on survival, should be carried out with caution due to differences in patient populations, PRRT protocols and response criteria.

#### 2.2.3. ^177^Lu-DOTATATE

The first prospective trials with ^177^Lu-DOTATATE were performed by the Rotterdam group in Erasmus Medical Center. Currently, the Rotterdam protocol of four cycles of 7.4 GBq ^177^Lu-DOTATATE per cycle, that was used in the NETTER-1 trial [70], is one of the most commonly used therapy regimens. Kwekkeboom et al. performed an efficacy analysis in 310 patients with GEP-NETs [47]. Patients received a cumulative activity up to 27.8–29.6 GBq (four intended cycles; treatment intervals 6–10 weeks). Radiological response assessment via SWOG was performed 3 months after the last administration of ^177^Lu-DOTATATE. The ORR was 46%, with a median PFS and OS of 33 and 46 months, respectively. Tumor uptake on baseline ^111^In-pentetreotide scintigraphy and a Karnofsky performance score (KPS) greater than 70 were independent predictors of tumor remission (complete response (CR), PR or minor response (MR)). Additionally, patients with progressive disease (PD) during response assessment had a significantly shorter survival, but there was no significant difference in survival between the patients with SD and tumor remission (CR, PR or MR) [47].

Further, Brabander et al. performed one of the largest retrospective studies with ^177^Lu-DOTATATE to date, in which they investigated the long-term efficacy, survival and safety in a Dutch patient cohort with predominantly low-grade GEP-NETs and bronchial NETs [45]. A subgroup of 443 patients treated with a cumulative activity of at least 22.2 GBq, was available for efficacy and survival analyses. An ORR of 39% was reached, and median PFS and OS were 29 and 63 months, respectively. Notably, patients with a primary NET of the pancreas had the longest OS of 71 months. A part of the patients in this analysis was also reported in the previously mentioned study by Kwekkeboom et al. [47], however in the current analyses a longer OS was seen in patients with PR or CR compared to those with SD [45], which was not the case in the study of Kwekkeboom et al. [47]. A likely explanation for this difference is that the current study used “best response” for response assessment compared to response assessment at 3 months after the last administration of ^177^Lu-DOTATATE in the Kwekkeboom study. In the 610 patients available for safety analysis, therapy-related myeloid neoplasm (t-MN) occurred in 2.1% of the patients [45]. 

A phase-II trial by Hamiditabar et al. included 143 patients and revealed an ORR of 8.4% and a DCR of 54.5% [49]. These relatively lower percentages in response rates compared to the previously described studies could be secondary to the fact that 45% of patients only received 1 to 3 cycles of approximately 7.4 GBq ^177^Lu-DOTATATE per cycle and differences in timing of response assessment. 

Furthermore, Ezziddin et al. investigated predictors of long-term outcome in 74 well-differentiated GEP-NET patients after treatment with ^177^Lu-DOTATATE [53]. A mean activity of 7.9 GBq per cycle (4 intended cycles; treatment intervals of 10–14 weeks) was administered. A Ki-67 index of more than 10%, KPS of less than or equal to 70, baseline NSE concentration greater than 15 ng/mL and baseline hepatic tumor burden of greater than or equal to 25% were independent predictors of a shorter OS. However, these results should be interpreted with caution given the retrospective nature of this study and subsequently retrospectively selected cutoff points [53]. To date, the only randomized controlled trial with ^177^Lu-DOTATATE is the phase-III NETTER-1 trial [70].

#### 2.2.4. NETTER-1 Trial

The NETTER-1 trial is a multicenter, randomized controlled trial comparing ^177^Lu-DOTATATE versus high dose SSAs in advanced midgut NET (*n* = 229) [70]. It has generated the strongest scientific evidence for the use of PRRT, as it is the only randomized PRRT trial that has reported its primary endpoint until now. The main inclusion criteria were: metastatic or inoperable, locally advanced midgut primary NET; RECIST 1.1-based disease progression in the three years before randomization, under a continuous regimen of SSA treatment (20 or 30 mg octreotide LAR per 3 to 4 weeks); KPS of at least 60; SSTR present on all tumoral lesions by ^111^In-pentetreotide scintigraphy; and Ki-67 index ≤ 20%. Main exclusion criteria were insufficient kidney, liver and hematological function and previous liver-directed therapy (surgery or transarterial therapy). Patients were randomized in a 1:1 ratio to the PRRT arm (*n* = 116), treated with 4 cycles of 7.4 GBq ^177^Lu-PRRT 8 weeks apart combined with 30 mg octreotide LAR q4 or to the control arm (*n* = 113) treated with 60 mg octreotide LAR q4.

The primary endpoint was PFS, with an estimated PFS at 20 months of 65.2% (95% confidence interval (CI): 50.0 to 76.8%) in the PRRT arm and 10.8% (95% CI: 3.5 to 23.0%) in the control arm, with a hazard ratio (HR) for progression or death of 0.21 (95% CI, 0.13 to 0.33; *p* < 0.001). The ORR according to RECIST 1.1 was 18% in the PRRT arm versus 3% in the control group (*p* < 0.001). The PRRT was well tolerated, with the most common adverse events being nausea in 65 patients (59%) and vomiting in 52 patients (47%), which in the vast majority was attributed to the nephroprotective amino acid infusion. Transient grade 3 or 4 neutropenia, thrombocytopenia, and lymphopenia according to common terminology criteria for adverse events (CTCAE) version 4.03 were observed in 1%, 2%, and 9% of patients, respectively, in the PRRT arm versus no patients in the control arm. No evidence of renal toxicity was seen among patients in the PRRT arm, but long-term follow-up is still warranted [70]. The recently presented final OS analysis revealed a median OS of 48 months in the ^177^Lu-DOTATATE group versus 36.3 months in the control group, which is clinically significant [71]. This difference was not statistically significant; this is most likely caused by a high rate (36%) of cross-over of patients in the control group to PRRT after progression.

This pronounced effect in tumor control was accompanied by a clinically meaningful effect on the quality-of-life (QoL) [72]. There was a substantial effect on time-to-deterioration (TTD) of QoL as assessed by the European Organisation for Research and Treatment of Cancer (EORTC) C-30 questionnaire, with a median TTD for global health status of 28.2 months vs. 6.1 months for the PRRT arm vs. the control arm, respectively (HR: 0.41, 95% CI: 0.24 to 0.69; *p* < 0.001). Effects of the same magnitude were observed for TTD for physical functioning (HR: 0.52; *p* = 0.015), pain (HR: 0.57; *p* = 0.025) and diarrhea (HR: 0.47; *p* = 0.011). Fifteen of 24 (63%) health-related domain scores were significantly different, and all HR were ≤ 0.86, all in favor of the PRRT arm. Furthermore, patient diaries demonstrated a substantial reduction in tumoral symptoms, such as abdominal pain (mean reduction 3.11 days per period of 28 days; *p* < 0.001), diarrhea (3.11 days; *p* = 0.0017) and flushing (1.98 days; *p* = 0.041) [73]. These data validate PRRT both as a tumor-controlling and symptom-relieving treatment.

Finally, analysis of outcome stratified by liver tumor burden (low <25%; moderate 25–50%; high >50%) showed that a high liver tumor burden was associated with a worse PFS in the control arm (median PFS of 9.1, 8.7, and 5.4 months for low, moderate, and high burdens, respectively; *p =* 0.017) [74]. However, there was no significant difference in PFS in these three groups in the PRRT arm (*p =* 0.72). The resulting HR for progression or death in these respective groups are 0.187 (*p* < 0.001), 0.216 (*p* = 0.0098) and 0.145 (*p* = 0.0018), all in favor of the PRRT arm. These data show that PRRT using ^177^Lu-DOTATATE is capable of neutralizing a major negative prognostic effect in metastatic NET patients. The absence of a large target lesion (defined as diameter > 30 mm) was associated with improved PFS (*p*  = 0.022), which raises the question if the longer-ranged ^90^Y-DOTATOC might have more beneficial effects in patients with such a lesion [74].

Based on the randomized data from the NETTER-1 trial [70] and the data of a large phase-II trial by the Rotterdam group [45], FDA- and EMA-approval was obtained for ^177^Lu-DOTATATE which has led to the commercialization of Lutathera^®^. The success of the NETTER-1 trial has been a beacon for the further development of novel radiopharmaceuticals targeting other molecular targets, e.g., ^177^Lu-PSMA-617 [29,30].

#### 2.2.5. Lung NET

The literature on PRRT consists of heterogenic patient cohorts, consisting of predominantly GEP-NETs. However, there are several studies that have evaluated PRRT solely in bronchial NETs. Well-differentiated bronchial NETs are classified into low-grade typical carcinoid and intermediate-grade atypical carcinoid [75]. Several clinical studies have shown that atypical carcinoids usually have high ^18^F-FDG uptake and low or moderate SSTR uptake and typical carcinoids usually have low FDG uptake and high STTR uptake. Zidan and Iravani et al. showed a wide inter- and intra-patient phenotypic heterogeneity on ^68^Ga-DOTATATE and ^18^F-FDG PET/CT in patients with bronchial NETs [76]. Around 50% of typical and atypical bronchial carcinoid patients had an unsuitable phenotype for PRRT, which was defined as patients with all lesions negative on both scans or patients with any SSTR negative/FDG positive lesions. This proportion is higher than what is seen in GEP-NETs. A prospective phase-II trial by Ianniello et al., investigated PRRT with ^177^Lu-DOTATATE in 34 patients with advanced bronchial carcinoids [67]. The median cumulative activity was 21.5 GBq (range 12.9–27.8 GBq). An ORR of 15% was achieved and a median PFS and OS of 18.5 and 48.6 months, respectively, was observed. Furthermore, Mariniello et al. retrospectively analyzed 114 patients with advanced bronchial carcinoids, treated with ^90^Y-DOTATOC, ^177^Lu-DOTATATE or a combination of ^90^Y-DOTATOC and ^177^Lu-DOTATATE [50]. An ORR of 26.5% was reached and a median PFS and OS of 28 and 58.8 months, respectively, was achieved. Notably, an objective response significantly delayed disease progression and prolonged survival in multivariate analyses. Even though it was initially thought that PRRT would be less effective in bronchial NETs due to the inter- and intra-patient heterogeneity on imaging with ^68^Ga-DOTATATE and ^18^F-FDG PET/CT, several studies have proven it to be effective in both efficacy and outcome and these results are comparable with studies done in GEP-NET patients. Dual-tracer imaging helps guide physicians in optimal patient selection in this subgroup of NETs [76].

### 2.3. Effect on Symptoms

The QoL in NET patients is lower compared to the general population and is mostly affected by tumoral mass effects and/or hormone production [77]. NETs can produce biogenic amines and peptides, which can induce a range of hormonal syndromes, e.g., serotonin production, leading to carcinoid syndrome and fibrosis [78,79,80]. The main symptoms of carcinoid syndrome include flushing, diarrhea and to a lesser extent bronchospasm. Abdominal pain in NETs is usually caused by tumor volume or intestinal ischemia, due to mesenteric lymph nodes and fibrosis [81]. The NETTER-1 trial has proven that PRRT is a symptom-relieving treatment [72,73]. Taking into consideration that all patients included in the NETTER-1 trial had radiological progressive disease at baseline, Zandee et al. retrospectively studied the effect of ^177^Lu-DOTATATE in low-grade metastatic midgut NETs with refractory carcinoid syndrome despite treatment with SSAs and without evidence of radiological disease progression at baseline [82]. Treatment with ^177^Lu-DOTATATE significantly reduced diarrhea and flushing, with a decrease in frequency from 6.1 ± 3.4 to 4.6 ± 3.6 per day (*p* = 0.009) and 4.3 ± 2.9 to 2.4 ± 2.7 per day (*p* = 0.002), respectively. These data substantiate the role of PRRT in the symptomatic treatment of carcinoid syndrome refractory to SSAs. Furthermore, around 10% of pancreatic neuroendocrine tumors (pNETs) are functional and secrete various hormones [83]. Functioning pNETs include insulinoma, glucagonoma, VIPoma or gastrinoma. These tumors have their own unique symptoms caused by the secretion of specific hormones. Zandee et al. investigated the effect of ^177^Lu-DOTATATE in the treatment of functioning pNETs in a cohort of 34 patients [68]. An ORR of 59% and a DCR of 78% were observed. In addition, 71% patients with uncontrolled syndrome-specific symptoms had a reduction of symptoms after PRRT. Hormonal crises occurred in 9% of the patients after PRRT, however this might be an underestimation given the fact that 50% (7/14) of the insulinoma patients were admitted for a glucose or octreotide infusion to prevent hypoglycemia [68]. Preventive intervention to minimize the occurrence of hormonal crises could be considered during PRRT.

## 3. Toxicity

Side-effects from PRRT are categorized in chronological order as acute (within hours), sub-acute (within days to weeks) and long-term (after years). PRRT is known to be well-tolerated, with limited toxicity. Acute side effects are nausea and vomiting, caused by the co-infusion of nephroprotective amino acids. Anti-emetic treatment can mostly control the nausea complaints [84]. Sub-acute effects are hematotoxicity, transient fatigue, tumor pain or low-grade hair loss (secondary to irradiation), which are commonly mild and self-limiting [70]. Furthermore, the occurrence of carcinoid crisis during PRRT is very rare and usually takes places after the first administration [85]. Long-term toxicity side-effects are mainly radiation nephropathy and persistent hematological dysfunction (PHD).

The kidneys are classically considered the main activity-limiting organ for PRRT. Renal irradiation is largely due to the glomerular filtration of the radiolabeled SSA with active proximal tubular reabsorption, subsequently leading to interstitial renal retention. Additionally, SSTR2 expression is present in the glomeruli and all SSTR subtypes are expressed in the renal tubuli. These two mechanisms lead to the total renal uptake of the radiolabeled SSA [86]. Renal impairment after PRRT is typically preceded by a latency period due to the slow cell turnover of kidney parenchyma. As such, (sub)acute nephrotoxicity after PRRT is rare and other causes should be investigated [87]. Long-term assessment of the renal function is of utmost importance. Patient-based risk factors for developing nephrotoxicity include baseline impaired renal function, hypertension, diabetes mellitus and previous nephrotoxic chemotherapy [88,89]. Furthermore, ^177^Lu-DOTATATE results in markedly lower renal toxicity compared to ^90^Y-DOTATOC, due to different characteristics regarding energy, tissue penetration, half-life and SSTR subtype affinity profiles (Table 1) [90,91]. In particular the more limited radiation range (2 vs. 11 mm) is thought to be responsible for the better kidney function outcome in patients treated with ^177^Lu-DOTATATE. A large retrospective study of 1109 patients treated with ^90^Y-DOTATOC reported permanent grade 4/5 nephrotoxicity in 9.2% of the patients [23], compared to the retrospective evaluation of 610 patients treated with ^177^Lu-DOTATATE in which no therapy-related long-term nephrotoxicity occurred [45]. Nephroprotection with co-infusion of an amino acid solution (containing L-lysine and L-arginine, alone or combined with other amino acids) during the administration of PRRT has led to a reduction of nephrotoxicity by inhibiting the megalin-mediated proximal tubular reabsorption [86].

In addition, patients with an impaired renal function can have a prolonged circulation of radiolabeled SSAs, which can lead to an increase in bone marrow irradiation and subsequently a higher grade of hematological toxicity [92]. Hematological toxicity manifests itself in two different forms, subacute myelotoxicity and late-term PHD. Subacute myelotoxicity, expressed as grade 3/4 hematological toxicity, occurs at comparable rates for ^90^Y-DOTATOC and ^177^Lu-DOTATATE in approximately 5–12% of patients and is self-limiting [23,70,89,93]. The nadir takes place around 4 to 6 weeks after administration, which has historically been an important factor for the 8-week interval between cycles in the Rotterdam/NETTER-1 regimen [70]. Depending on the severity and type, activity reduction and/or treatment delay can be performed. There is no increased risk in patients exposed to prior targeted agents [94]. Infectious complications are rarely seen, with a very low risk for neutropenic fever, even though lymphocytopenia is a frequent side-effect in patients receiving PRRT. This is explained by mainly the B-cell subpopulation being targeted by PRRT due to an overexpression of SSTR2, however the risk of opportunistic infections seems to be related to the T- and Natural Killer-cells, which are largely unaffected by PRRT [90]. As such, lymphocytopenia should not lead to changes in therapy. Myelotoxicity appearing soon in the treatment course is a risk factor for PHD and injected activity modification due to a higher risk of developing long-term myelotoxicity could be envisioned [95].

The most severe long-term side-effect of PRRT is the development of PHD, either in the form of bone marrow failure/aplasia or a t-MN. PHD after PRRT is rare, but is associated with a detrimental impact on QoL and a poor prognosis after t-MN diagnosis. According to the 2016 World Health Organization classification, t-MN includes myelodysplastic syndrome and acute myeloid leukemia in patients previously exposed to cytotoxic therapy [96]. The origin of radiation-induced t-MN is very intricate and includes the formation of single or double strand breaks in the DNA, which can eventually lead to genetic mutations, including loss of function or oncogene activation [89]. Studies including large datasets, report an incidence of PHD after PRRT between 1.8 and 4.8%, with a median latency of 41 months [89,97,98,99]. Brieau et al. reported an incidence of t-MN of 20% after PRRT, in a limited subgroup of 20 patients heavily pretreated with alkylating chemotherapy [100]. The high incidence of t-MN in their study is presumably caused partially by a direct effect of the prior alkylating chemotherapy, which is known to induce myeloid neoplasms [84]. Further, the latency period from the first cycle of PRRT to t-MN diagnosis is variable, ranging from several months to over 10 years, and may be shorter in patients receiving concurrent radiosensitizing chemotherapy during PRRT [89,97,98,99]. However, t-MN patients have a poor prognosis reflected by a median OS of 13 months after diagnosis of t-MN, reported by both Goncalves et al. and Chantadisai et al. [97,98]. Subsequently, several attempts have been made to define potential causal risk factors of t-MN in patients receiving PRRT, however, no significant consistent risk factors have emerged across studies [89,99]. It is hypothesized that this absence of causal risk factors can be secondary to intrinsic genetic factors leading to individual differences in susceptibility of radiation induced effects [89,97].

## 4. Optimization

### 4.1. Dose-Effect Concept and Individualized Dosimetry

At present, ^177^Lu-DOTATATE is the main radiopharmaceutical used for PRRT. The Rotterdam/NETTER-1 protocol for ^177^Lu-DOTATATE consists of 7.4 GBq per cycle/4 cycles/8-week interval and is in widespread use for PRRT, even though it is known that there is an intra- and interindividual variability in absorbed radiation doses in metastases and critical organs for the same administered activity [62,70]. The kidneys and bone marrow are classically considered the main activity-limiting organs for PRRT and the cumulative activity given per patients is conceptually restricted by the maximum acceptable absorbed doses to these organs. Commonly, the accepted absorbed dose limits for the kidneys and bone marrow are set at 23 Gy and 2 Gy, respectively, which were initially adopted from experience with external beam radiation therapy (kidneys) and clinical studies with ^131^I in patients with thyroid cancer (bone marrow) [86,101]. However, these dose limits are under debate and it is suggested that higher absorbed doses can be administered without a large increase in toxicity [93]. In addition, the extrapolation of the absorbed dose limits to the kidneys from external beam radiation therapy to PRRT is questionable, given the differences in radiobiology, and might be different for different radionuclides (^90^Y vs. ^177^Lu; β^-^ vs. α-emitter). To counter these differences, the linear-quadratic radiobiological model is applied to convert the absorbed dose to the BED [102]. Bodei et al. proposed the BED upper limits for the kidneys to be 28 Gy and 40 Gy, respectively for patients with and without risk factors [103].

The Uppsala group has evaluated the dose-response concept in pNETs and SI-NETs, in which the relation between the absorbed tumor dose and treatment response is investigated. A significant correlation between the absorbed tumor dose and tumor size reduction was established in 24 lesions of 24 patients (one lesion per patient was studied) with metastatic pNETs (Pearson correlation coefficient (R^2^) of 0.64 for tumors > 2.2 cm and 0.91 for the subgroup of tumors > 4.0 cm) [104]. The better correlation in larger tumors might be due to lower systematic error in image-based dose estimation. On the contrary, no tumor dose-response relationship was found in 25 metastases from 25 SI-NET patients (one lesion per patient was studied) [105]. However, a correlation was found between tumor volume shrinkage and administered radioactivity (r^2^ = 0.25, *p* = 0.01), and between tumor diameter reduction (RECIST 1.1) and administered radioactivity (r^2^ = 0.28, *p* = 0.01). Further larger studies are needed to investigate the dose-response concept.

**Figure 2:**
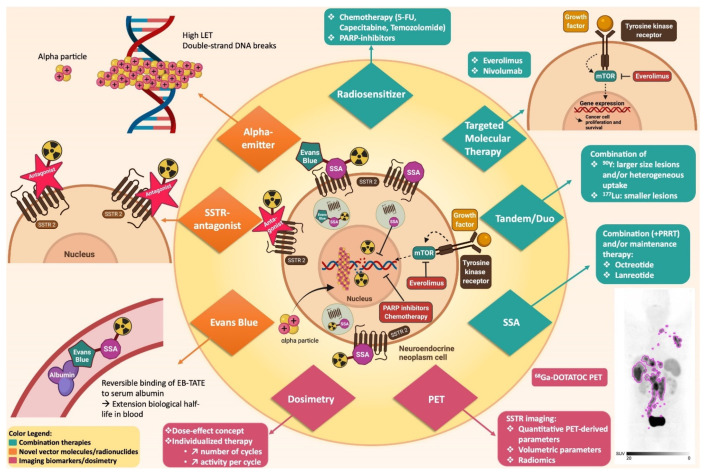
Optimization of PRRT. LET = linear energy transfer; SSTR = somatostatin receptor; SSA = somatostatin analogue; PRRT = peptide receptor radionuclide therapy; PARP = Poly-[ADP-ribose]-polymerase; mTOR = mammalian target of rapamycin; 5-FU = 5-fluorouracil; PET = positron emission tomography.

Moreover, the use of individualized dosimetry to determine the maximum tolerable administered activity on an individual level has been investigated. Two different strategies have been described using ^177^Lu-DOTATATE, firstly increasing the number of cycles while maintaining 7.4 GBq/cycle and secondly increasing the activity per cycle. The first method was used by Garske-Roman et al., who investigated the impact of a dosimetry-guided study protocol on outcome and toxicity in 200 patients with advanced NETs [48]. Cycles of 7.4 GBq ^177^Lu-DOTATATE were repeated until an absorbed dose of 23 Gy was reached in the kidneys or until other reasons were present to stop the therapy. The dose limit to bone marrow was set at 2 Gy, but this was not reached in any patient, making the kidneys the main activity-limiting organ. Most patients (68.5%) received more than 4 cycles to reach an absorbed dose of 23 Gy to the kidneys. Median PFS and OS were significantly longer in patients who reached 23 Gy to the kidneys compared to those who did not (median PFS: 33 vs. 15 months and median OS: 54 vs. 25 months). No major nephrotoxicity related to the treatment was observed [48]. Another trial exploring this strategy is the phase-II ILUMINET trial, in which cycles of 7.4 GBq were repeated until a renal BED of 27 Gy or 40 Gy was reached, respectively for patients with or without renal or hematological risk factors [106]. In an interim analysis, more than 4 cycles could be administered in 73% of the patients and no grade III/IV nephrotoxicity was observed. Long-term results of this trial are awaited given the latency period for nephrotoxicity after PRRT. The second method for individualized dosimetry was implemented in the P-PRRT trial [62], in which the injected activity was personalized according to the glomerular filtration rate and body surface area for the first cycle and renal dosimetry for the subsequent cycles. A median 1.26-fold increase (range 0.47–2.12 fold) in cumulative maximum tumor absorbed dose was established with this method compared to a simulation of the fixed injected activity method of 7.4 GBq/cycle. PR, MR and SD was achieved in 23%, 36% and 33% of patients, respectively. Subacute grade III/IV hematotoxicity occurred in less than 10% of patients which is comparable with the Rotterdam/NETTER-1 regimen and no severe nephrotoxicity was reported [62]. In conclusion, using individualized dosimetry as a tool to adapt the total administered activity or the number of therapy cycles holds potential as an optimized strategy compared to the current standard NETTER-1/Rotterdam protocol and could be more often used in clinical practice in the future (Figure 2). Several trials are ongoing to further investigate this concept (NCT03454763, NCT04917484).

### 4.2. Combination Therapies

#### 4.2.1. Tandem and Duo PRRT

Tandem and duo PRRT consists of a combination of the high-energy ^90^Y beta-emitter for targeting lesions with a larger size and/or heterogeneous uptake (with more crossfire effect), and the medium-energy ^177^Lu beta/gamma-emitter for targeting smaller lesions (with a higher fraction of the total energy deposited within the tumor itself, and not in the surrounding tissue). Theoretically, a synergistic effect can be achieved by combining these two radionuclides with different absorption properties. In general, tandem PRRT is defined by co-administration of a mixture of ^90^Y/^177^Lu-DOTATATE/-TOC, or infusion of both radiopharmaceuticals on the same day. Duo PRRT consists of alternating cycles of ^90^Y-DOTATATE/-TOC and ^177^Lu-DOTATATE/-TOC. Kunikowska et al. performed a first-in-human, non-randomized study comparing monotherapy with ^90^Y-DOTATATE (*n* = 25) with tandem ^90^Y/^177^Lu-DOTATATE (*n* = 25) in patients with progressive advanced NETs [107]. A significant increase in OS was achieved in the tandem group compared to the monotherapy group (median OS not reached vs. 26 months, after a median of 34.6- and 37.7-months follow-up, respectively, *p* < 0.027), but these results are hypothesis-generating only as there can be differences in the patient populations treated. No benefit in objective response was achieved between the two groups [107]. Further, several tandem PRRT studies have revealed response and PFS results that are comparable with monotherapy PRRT studies (Table 2) [51,59,108]. Moreover, a cohort study with 486 patients showed that the group receiving alternating cycles of ^90^Y-DOTATOC and ^177^Lu-DOTATOC (n = 249) had a significantly longer survival compared to the group receiving ^90^Y-DOTATOC monotherapy (*n* = 237) (5.51 vs. 3.96 years, respectively, HR = 0.64, *p* = 0.006) [109]. Further, Radojewski et al. showed that a combination of ^90^Y-DOTATOC and ^177^Lu-DOTATOC was associated with an increase in survival compared to ^90^Y-DOTATOC monotherapy (66.1 vs. 47.5 months, respectively, *p* < 0.001) or ^177^Lu-DOTATOC monotherapy (66.1 vs. 45.5 months, respectively, *p* < 0.001) [110]. Moreover, in the study of Baum et al., 1048 patients were included and received PRRT with either ^177^Lu monotherapy (36%), ^90^Y monotherapy (15%) or a combination of ^90^Y and ^177^Lu in tandem or duo (49%) [111]. In multivariate analyses, a significant difference in OS was seen in favor of tandem/duo PRRT (mOS for tandem/duo PRRT 64 months, ^177^Lu 44 months and ^90^Y 24 months; HR = 1.67 for ^177^Lu monotherapy and 2.89 for ^90^Y monotherapy). In conclusion, even though efficacy and PFS are comparable between tandem/duo PRRT and PRRT monotherapy, there is evidence suggesting a survival benefit in favor of tandem/duo PRRT. Further randomized controlled trials (RCTs) will have to demonstrate the superiority of this concept before widespread clinical adoption can occur, to remove logistic and regulatory hurdles, as ^90^Y-DOTATOC is currently not authorized by FDA nor EMA.

#### 4.2.2. ChemoPRRT

An emerging strategy leading to a change in the PRRT paradigm, is the addition of radiosensitizing chemotherapy (Table 3). The goal is to enhance the treatment efficacy and outcome without a substantial increase in toxicity. Several clinical studies have been published combining PRRT with 5-fluorouracil (5-FU), capecitabine or temozolomide. 5-FU is a cytotoxic agent belonging to the class of fluorinated pyrimidines and is administered intravenously. Capecitabine, a prodrug of 5-FU, has the additional advantage that it can be administered orally [112]. Temozolomide is an alkylating agent and causes DNA methylation injury. It is proposed that tumor sensitivity to temozolomide is dependent of the levels of DNA repair enzyme O^6^-methylguanine DNA methyltransferase (MGMT). Both MGMT deficiency as well as treatment response to temozolomide is more commonly observed in pNETs than lung or SI-NETs, which is explained by the higher levels of MGMT deficiency in pNETs [113]. A synergistic effect is apparent when combining capecitabine and temozolomide, most likely due to the depletion of MGMT caused by capecitabine, which strengthens the effect of temozolomide. This is the reason why the treatment regimens add temozolomide after substantial exposure to capecitabine in the clinical studies described below [114]. 

A phase-II study investigated the combination of ^177^Lu-DOTATATE with capecitabine in 33 patients with progressive metastatic well-differentiated NETs [115]. Mild hematotoxicity was seen, which included transient grade 3 thrombocytopenia in a single patient (3%). A high DCR of 94% was achieved. Median PFS and OS were not reached at the time of the analysis [115]. Hereafter, given the known synergistic effect of combining capecitabine with temozolomide, a phase-I-II study was performed combining ^177^Lu-DOTATATE with capecitabine-temozolomide (CAPTEM) in 34 advanced low-grade NETs [116]. Hematotoxicity was mild with grade 3 neutropenia in 6% of the patients, however there were no episodes of febrile neutropenia. An ORR of 53% was observed with a median PFS of 31 months. Long-term follow-up of the hematotoxicity of these two studies [115,116] revealed that the association of PRRT with CAPTEM causes a modest and reversible hematotoxicity which is not significantly greater compared to treatment with PRRT alone [117]. Furthermore, the same group performed a phase-II study investigating the combination of CAPTEM with ^177^Lu-DOTATATE in 30 patients with advanced low-grade pNET [118]. Adverse effects were limited and a high ORR of 80% was achieved with a median PFS of 48 months. Kong et al. retrospectively assessed predictors of response and OS in 63 grade 1–2 NET patients treated with concurrent ^177^Lu-DOTATATE and 5-FU [119]. DCR was 68% and median OS was not estimable at a median follow-up of 60 months. An objective response was significantly associated with a longer OS in univariate analysis. Patients with a primary pancreatic site and lesions larger than 5 cm had significantly lower ORRs in univariate analysis, which can indicate that an aggressive treatment approach is needed for these patients [119]. Furthermore, a 2-arm cohort analysis compared concomitant ^177^Lu-DOTATATE and capecitabine (*n* = 88) with ^177^Lu-DOTATATE monotherapy (*n* = 79) and revealed a significant lengthening of OS in the ^177^Lu-DOTATATE and capecitabine group compared to ^177^Lu-DOTATATE monotherapy group (median OS not reached vs. 48 months, respectively, after a mean follow-up of 32.4 months; *p* = 0.0042) [120]. 

**Figure 3:**
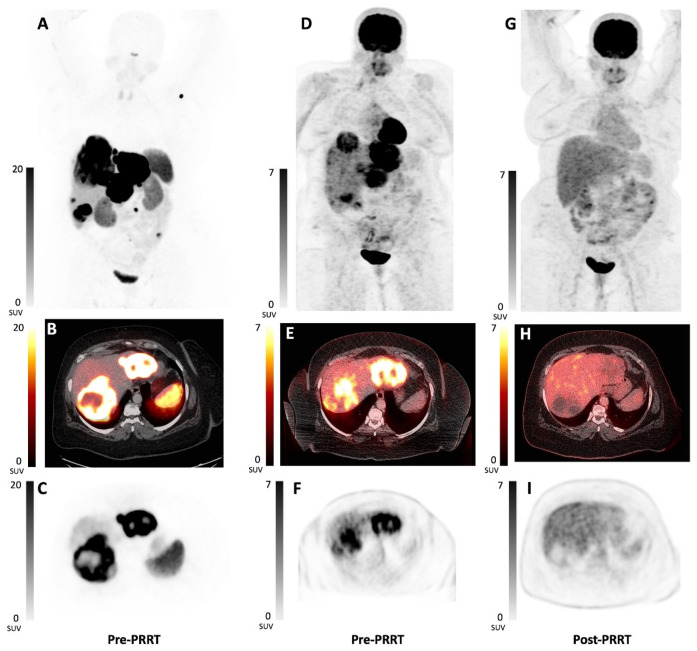
Example of FDG positive disease and response after 2 cycles PRRT. Thirty-two-year-old patient with an advanced neuroendocrine tumor of the small intestine (Ki-67 index: 10%) that presented with disease progression after previous treatment with somatostatin analogues, everolimus and temozolomide-capecitabine. She was deemed eligible for peptide receptor radionuclide therapy (PRRT) after work-up. ^68^Ga-DOTATATE PET/CT scan prior to PRRT ((**A**) maximum intensity projection (MIP) image; (**B**) fusion PET/CT; (**C**) native PET) showed strongly increased somatostatin receptor-expression in the malignant bone, lymph nodes and liver metastases. ^18^F-FDG PET/CT prior to PRRT ((**D**) MIP image; (**E**) fusion PET/CT; (**F**) native PET) showed strong ^18^F-FDG-avidity in the liver metastases. ^18^F-FDG PET/CT after 2 cycles of PRRT revealed a complete metabolic response in the liver metastases ((**G**) MIP image; (**H**) fusion PET/CT; (**I**) native PET). SUV = standardized uptake value.

Another population of interest for peptide receptor chemoradionuclide therapy (PRCRT) are the highly proliferating NETs characterized by tumor dedifferentiation, higher tumor grade, worse OS outcome and most commonly ^18^F-FDG-avidity of the tumor lesions. ^18^F-FDG PET positivity in NETs is associated with a poor survival and shorter PFS after PRRT, in comparison with FDG-negative disease (Figure 3) [121,122]. Kashyap et al. retrospectively investigated PRCRT (combination of ^177^Lu-DOTATATE and 5-FU) in 52 patients with ^18^F-FDG-avid disease and the majority having grade 2 advanced NETs [123]. In their institution, ^18^F-FDG PET/CT was performed as baseline evaluation in patients with Ki-67 > 5%, disease progression in less than 6 months or malignant lesions without SSTR expression. A high DCR of 98% was achieved and 27% of the patients achieved complete metabolic response on ^18^F-FDG PET/CT despite having residual SSTR-avid disease, most likely due to the eradication of the dedifferentiated lesions by PRCRT. Additionally, it was expected that the prognosis in this patient cohort would be poor, however median PFS of 48 months was achieved and median OS was not reached during a median follow-up time of 36 months. Toxicity was low, despite 67% of the patients having received prior chemotherapy [123]. Moreover, a prospective phase-II study was performed using a combination of ^177^Lu-DOTATATE and metronomic capecitabine in 37 patients with advanced SSTR- and ^18^F-FDG-positive grade 1 to 3 GEP-NETs [124]. High response rates were reported, and toxicity was acceptable. A median PFS of 31.4 months was observed, which may suggest a benefit of PRCRT in this population of ^18^F-FDG-positive GEP-NETs compared to PRRT monotherapy. Results of RCTs with PRCRT are eagerly awaited. To date, only preliminary results of the phase-II “CONTROL NET” RCT have been presented [125]. This trial compares a combination of ^177^Lu-DOTATATE with CAPTEM (experimental arm) versus ^177^Lu-DOTATATE monotherapy (control arm) in patients with low to intermediate midgut NETs. Forty-seven patients were included. The 15-month PFS was 90% versus 92% and ORR was 25% versus 15% for PRRT plus CAPTEM versus PRRT monotherapy, respectively. However, grade 3/4 toxicity occurred more frequently in the PRRT plus CAPTEM arm [125]. Further follow-up is awaited to determine the benefit of combining PRRT and CAPTEM and initiate a phase-III trial.

**Table 3:** ChemoPRRT.

First Author	Design	*n*	Subtype	Setting	Compound	Chemo	ORR	DCR	CR	PR	MR	SD	PD	Criteria	Median PFS (mo)	Median OS (mo)	Grade 3/4 Hemato-Toxicity	Comments
Ballal [120]	R	88	Grade 1-2-3 GEP-CUP-other-NET/neural crest	Imaging progression/Biochemical progression	^177^Lu-DOTATATE	capecitabine	43%	93%	0%	34%	9.1%	50%	6.8%	RECIST 1.1	NR	NR	1%	
Kong [119]	R	63	Grade 1-2 GEP-lung-thymus-CUP-NET	Biochemical or imaging progression > Uncontrolled symptoms	^177^Lu-DOTATATE	5-FU	39%	68%	0%	30%	9%	29%	32%	RECIST 1.1	NA	NR	NA	63 of the 68 included patients received chemoPRRT (response rates did not differentiate between pt receiving monotherapy PRRT and chemoPRRT)
Kashyap [123]	R	52	Grade 1-2-3 GEP-CUP-NET	Imaging or biochemical progression/Uncontrolled symptoms	^177^Lu-DOTATATE	5-FU	30%	98%	2%	28%	-	68%	2%	RECIST 1.1	48	NR	6%	FDG-positive disease
Nicolini [124]	P	37	Grade 1-2-3 GEP-NET	Progressive metastatic or inoperable NETs	^177^Lu-DOTATATE	capecitabine	30%	85	0%	30%	-	55%	15%	RECIST 1.1	31	NR	16%	FDG-positive disease
Claringbold [116]	P	34	Well-differentiated GEP-lung NET	Imaging progression/highly advanced metastatic disease and substantial symptoms	^177^Lu-DOTATATE	CAPTEM	53%	91%	15%	38%	-	38%	9%	RECIST 1.1	31	NR	6%	Predominantly grade 1 NETs
Claringbold [115]	P	33	Well- or moderately differentiated EP-lung-CUP NET	Imaging progression	^177^Lu-DOTATATE	capecitabine	24%	94%	0%	24%	-	70%	6%	RECIST 1.1	NR	NR	3%	
Claringbold [118]	P	30	Grade 1-2 pNET	Imaging progression	^177^Lu-DOTATATE	CAPTEM	80%	100%	13%	67%	-	20%	0%	RECIST 1.1	48	NR	10% TBC 10% RBC	

PRRT = peptide receptor radionuclide therapy, *n* = number of included patients, ORR = objective response rate, DCR = disease control rate, CR = complete response, PR = partial response, MR = minor response, SD = stable disease, PD = progressive disease, PFS = progression-free survival, OS = overall survival, mo = months, P = prospective, R = retrospective, NET = neuroendocrine tumor, GEP = gastroenteropancreatic, CUP = unknown primary tumor, pNET = pancreatic NET, EP = enteropancreatic, RECIST = Response Evaluation Criteria in Solid Tumors, NA = not available, NR = not reported, 5-FU = 5-fluorouracil, CAPTEM= capecitabine-temozolomide, pt = patients, FDG = fluorodeoxyglucose, TBC= thrombocytopenia, RBC = anemia.

#### 4.2.3. Targeted Molecular Therapy

Everolimus is a mammalian target of rapamycin (mTOR) inhibitor. Cell growth, proliferation and survival are regulated by this mTOR pathway and this pathway is often deregulated in cancer [126]. The phase-I NETTLE study performed a proof-of-concept study by combining ^177^Lu-DOTATATE with everolimus, in order to establish an optimal safe dose of everolimus [127]. Sixteen patients with advanced progressive well-differentiated GEP-NETs were included. An ORR of 44% was achieved. Hematotoxicities were apparent at the 3 dose levels of everolimus studied (5, 7.5 and 10 mg), but were manageable and reversible. Nephrotoxicity was the dose-limiting factor, leading to the maximum tolerated dose of 7.5 mg everolimus in combination with PRRT [127].

Further, the combination of PRRT with the immune checkpoint inhibitor nivolumab has recently been explored in a phase-I study including nine patients with advanced lung NENs (six small cell lung cancer, two atypical bronchial carcinoid and one high-grade neuroendocrine carcinoma) [128]. Dose level 1 consisted of ^177^Lu-DOTATATE 3.7 GBq (8-week interval, 4 cycles intended) plus nivolumab 240 mg (2-week interval), and dose level 2 consisted of ^177^Lu-DOTATATE 7.4 GBq (8-week interval, 4 cycles intended) plus nivolumab 240 mg (2-week interval). Only one dose-limiting toxicity, consisting of a grade 3 rash, was noted in one patient being treated at dose level 2. Grade 3 treatment-related adverse events were noted in 56% (5/9) of the patients: lymphopenia (*n* = 4), rash (*n* = 1), pneumonitis (*n* = 1), anemia (*n* = 1) and thrombocytopenia (*n* = 1). There were no grade 4 of 5 adverse events [128]. Additional studies are awaited to further evaluate the treatment efficacy and safety of this combination therapy. 

#### 4.2.4. SSA

The RCTs PROMID and CLARINET have shown that SSAs have an antiproliferative effect in metastatic enteropancreatic NETs, which is reflected by a significant increase in PFS [8,9,129]. However, a benefit in OS has not been described in these studies, probably due to crossover from the placebo group to the SSA group. At present, PRRT studies include a heterogeneous patient population with patients using short-acting SSAs and/or long-acting SSAs during PRRT and maintenance SSAs after PRRT. The reason for this heterogeneity is that a large portion of patients have carcinoid syndrome and/or functioning NETs, and SSAs can counter these symptoms while waiting for the efficacy of PRRT to kick in or as maintenance after completion of PRRT. The effect of long-acting SSAs on ^68^Ga-DOTATATE PET has been investigated in a few prospective studies. Injection of long-acting SSAs prior to ^68^Ga-DOTATATE PET did not decrease tumor uptake, however, uptake in normal organs was decreased leading to an increased tumor-to-liver ratio [130,131]. This is in contrast to the current guidelines that still suggest to cease long-acting SSAs 3–4 weeks prior ^68^Ga-DOTATATE PET [132]. However, these results cannot be projected to the effect of SSAs on uptake of PRRT, keeping in mind that on average a 10 times higher peptide amount is used for PRRT compared to ^68^Ga-DOTATATE PET [131]. The NETTER-1 RCT compared the combination of PRRT with octreotide LAR 30 mg every 4 weeks to octreotide LAR 60 mg every 4 weeks and demonstrated a marked increase in PFS and ORR [70]. As there was no arm with PRRT alone, the addition of SSA in non-functional NETs has not been studied yet by a properly powered RCT. One study by Yordanova et al. retrospectively investigated the effect on survival of adding SSAs to PRRT (combination therapy and/or maintenance therapy) in advanced GEP-NET patients [133]. Compared to the PRRT monotherapy group, patients in the SSA plus PRRT group had a significant improvement in PFS (median PFS 27 vs. 48 months, *p* = 0.012, respectively), OS (median OS 47 vs. 91 months, *p* < 0.001, respectively) and ORR (40% vs. 63%, *p* = 0.008, respectively). Patients with Ki-67 ≥ 10%, high tumor burden and functioning tumors showed the most significant benefit in survival in the SSA plus PRRT group. Future RCTs are needed to further investigate the effectiveness of adding SSAs to PRRT in non-functional tumors.

#### 4.2.5. Radiosensitizers

Poly-[ADP-ribose]-polymerase 1 (PARP-1) activity is required for the DNA damage repair of single strand breaks that can be caused by PRRT. When single-strand breaks are not repaired, they will lead to replication fork arrest and double-strand break formation during replication, and ultimately to cell death. PARP inhibitors are currently used in the treatment of several solid tumors (e.g., ovarian cancer, breast cancer, adenocarcinoma of the pancreas, prostate cancer). As such, an increase in preclinical research using PARP inhibitors as a radiosensitizer has emerged over the last few years. Several preclinical studies have shown that the combination of PARP inhibitors with ^177^Lu-DOTATATE leads to increased cell death [134,135,136]. A phase-I dose-escalation study combining talazoparib with ^177^Lu-DOTATATE in patients with metastatic pNETs has started recently (NCT05053854).

### 4.3. Novel Vector Molecules and Radionuclides

#### 4.3.1. Somatostatin Receptor Antagonists

Over the last few decades, the PRRT-paradigm for effective tumor targeting consisted of using receptor agonists which internalize after receptor binding, hereby causing tracer accumulation in the tumor cells. However, SSTR-antagonists are slowly emerging in the SSTR imaging and PRRT scene, with several preclinical studies showing their superiority over SSTR-agonists, despite the very slight amount of internalization [15]. It is postulated that SSTR-antagonists, compared to SSTR-agonists, have a higher number of receptors with a favorable configuration of their binding site, despite similar affinity profiles [137,138]. In vitro and in vivo preclinical studies comparing the SSTR2-antagonist ^177^Lu-OPS201 (also referred to as ^177^Lu-DOTA-JR11, ^177^Lu-IPN01072 or ^177^Lu-satoreotide tetraxetan) to ^177^Lu-DOTATATE, have shown that ^177^Lu-OPS201 exhibits a higher tumor uptake, higher number of double-strand breaks, longer tumor residence time and improved tumor-to-kidney dose ratio [139,140]. A pilot study with ^177^Lu-OPS201 was performed in four patients with advanced neuroendocrine neoplasms (NENs) and chronic grade 2 or 3 kidney disease [141]. Compared to ^177^Lu-DOTATATE, ^177^Lu-OPS201 showed a longer tumoral residence time and higher tumor uptake resulting in 1.7–10.6 times higher tumor doses. Toxicity was minor and reversible. Moreover, a phase-I trial including 20 patients with progressive well-differentiated NETs using ^177^Lu-OPS201 led to a high ORR of 45% and SD in 40% of patients [142]. Nephrotoxicity was not observed, however rather unexpectedly, grade 4 hematological toxicity occurred in 4 of 7 patients after the second cycle causing the protocol to be modified to limit the cumulative absorbed bone marrow dose to 1 Gy. Recently, the Bad Berka group reported the first-in-human study with the SSTR antagonist ^177^Lu-DOTA-LM3 in 51 patients with advanced NENs [143]. Sixty-nine percent of the patients were previously treated with ^177^Lu-DOTATOC or -TATE. Promising results were achieved with a high DCR of 85%. The occurrence of hematological toxicity was low, contrary to the previously described phase-I trial with ^177^Lu-OPS201 [142]. This is most likely due to a different molecular structure and peptide amounts. Noteworthy, 37 patients had no or low SSTR2 agonist binding on baseline ^68^Ga-DOTATOC or -TATE PET/CT (insufficient for agonist PRRT) [143]. Several prospective trials with SSTR antagonists are ongoing.

#### 4.3.2. Evans Blue

An attempt to improve the pharmacokinetics of the known radiolabeled SSAs was made by conjugating an Evans blue analog onto octreotate (EB-TATE). This results in a reversible binding of EB-TATE to serum albumin through the EB moiety, hereby extending its biological half-life in blood [144]. The first-in-human study with a single dose of ^177^Lu-DOTA-EB-TATE was conducted in five patients with advanced metastatic NETs [145]. In comparison to ^177^Lu-DOTATATE (*n* = 3 patients), ^177^Lu-DOTA-EB-TATE achieved an extended blood-circulation and a 7.9-fold increase in tumor dose delivery. No adverse effects were noticed which was excepted as the administered activity was subtherapeutic, however, the dose delivery to the kidneys and bone marrow was significantly higher in patients receiving ^177^Lu-DOTA-EB-TATE compared to ^177^Lu-DOTATATE (3.2 and 18.2-fold, respectively) [145]. These findings led to a dose escalation study in 33 patients with metastatic NETs [146]. A significant decrease in maximum standardized uptake value (SUV_max_) after treatment in the ^177^Lu-DOTA-EB-TATE group was achieved compared to the ^177^Lu-DOTATATE group (mean ΔSUV_max_% = −19.0 ± 21.5 and 8.4 ± 48.8, respectively, *p* = 0.045), in a selection of lesions with a comparable baseline SUV_max_ (range 15–40). Furthermore, the safety and efficacy of administering up to three cycles ^177^Lu-DOTA-EB-TATE has been evaluated in 32 NET patients (three groups; median cumulative activity 3.5 GBq, 5.7 GBq and 10.5 GBq) [147]. Hematotoxicity was acceptable and no nephrotoxicity occurred, but only a short follow-up period after the last cycle of PRRT was observed. Response assessment results were promising, but again, based on a decrease in SUV (EORTC and modified Positron Emission Tomography Response Criteria in Solid Tumors (PERCIST)). Recently, an intraindividual comparison of the pharmacokinetics of ^177^Lu-DOTA-EB-TATE and ^177^Lu-DOTATOC was conducted in 5 patients with progressive SSTR positive disease [148]. The ratio of absorbed doses in tumors and critical organs was not superior for ^177^Lu-DOTA-EB-TATE compared to ^177^Lu-DOTATOC. Therefore, all 5 patients were eventually treated with ^177^Lu-DOTATOC. The higher tumor dose delivered with this agent comes at the price of an even higher increase of the bone marrow dose, and thus it remains uncertain if using this compound is superior to higher activity/more cycles of established radiopharmaceuticals. More prospective studies are needed to evaluate the toxicity/treatment efficacy balance of this new compound.

#### 4.3.3. Alpha-Emitters

The use of alpha-emitters (such as lead-212, actinium-225 and its daughter bismuth-213) in PRRT is an emerging strategy, however, the availability of these alpha-emitters is still limited world-wide. To date, preclinical and clinical research studies with actinium-225 or bismuth-213 were predominantly conducted by the extraction of actinium-225 from thorium-229 sources, arising from the decay of fissile uranium-233. However, these thorium-229/uranium-233 stocks are limited due to legal requirements related to fissile materials. This led to the investigation of several accelerator-based production routes over the last years, which will increase the availability of these alpha-emitters in the future [149]. 

A first-in-human study was conducted by administering ^213^Bi-DOTATOC to eight patients with progressive NETs refractory to nonradioactive octreotide and tandem therapy with ^90^Y/^177^Lu-DOTATOC (*n* = 7 intra-arterial administration into the common hepatic artery; *n* = 1 systemic administration) [25]. ^213^Bi-DOTATOC was able to overcome resistance against beta radiation and induce long-term tumor remission. Nephrotoxicity and acute hematotoxicity were in the acceptable range. A prospective study was performed with ^225^Ac-DOTATATE in 32 patients with metastatic GEP-NENs who had SD after completing ^177^Lu-DOTATATE (*n* = 14) or PD on ^177^Lu-DOTATATE therapy (n = 18) [28]. The mean cumulative radioactivity administered was 22,550 ± 9842 kBq (range 7770–44,400 kBq). A planned interim analysis of morphological response conducted 8 weeks after the second cycle revealed PR in 62.5%, MR in 25% and SD in 12.5% of the 24 assessed patients. The morphological response assessment in the patient group who had SD after completing ^177^Lu-DOTATATE, revealed PR (*n* = 8) or MR (*n* = 4) in the 12 patients who were assessed. In addition, in the patient group that had PD on ^177^Lu-DOTATATE, no PD was seen during the response assessment after two cycles of ^225^Ac-DOTATATE. No grade III/IV hematotoxicity, nephrotoxicity or hepatotoxicity occurred [28]. Furthermore, preliminary results of the first-in-human dose-escalation study with ^212^Pb-DOTAMTATE in 20 patients are promising [27]. At present, 6 of 10 patients, having received ^212^Pb-DOTAMTATE at the highest dose level of 2.50 kBq/kg/cycle, have completed all four cycles of treatment. A high ORR of 83.3% (5/6 patients) via RECIST 1.1 was achieved and toxicity was low. In conclusion, these preliminary clinical results provide proof-of-principle evidence that α-PRRT can overcome resistance to β-PRRT.

### 4.4. Administration through the Hepatic Artery

**Figure 4:**
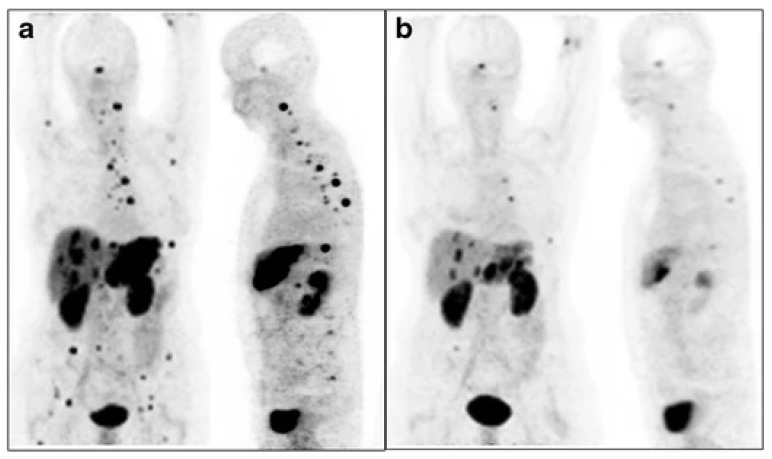
A patient with an extensive tumor burden in the left liver lobe and multiple lesions in the right lobe and disseminated bone marrow metastases predominantly in the spine and pelvis ((**a**) coronal and sagittal maximum-intensity projections ^68^Ga-DOTATOC PET). Liver metastases showed significant shrinkage after administration of 10.5 GBq of ^213^Bi-DOTATOC into the common hepatic artery (**b**). Additional systemic efficiency resulting from the ^213^Bi-DOTATOC reaching the systemic circulation after the first pass of the liver was noted after 6 months in that most of the bone marrow metastases had also diminished (**b**). This image nicely demonstrates the potential of alpha-emitters and the feasibility of intra-arterial administration of peptide receptor radionuclide therapy. This image was originally published by Kratochwil et al. [25] and it is licensed under a Creative Commons Attribution 4.0 International License (https://creativecommons.org/licenses/by/4.0/, accessed on 20 October 2021). No adaptations to this image were made.

Given the fact that NETs often metastasize to the liver, it can be hypothesized that intra-arterial (IA) administration of the radiopharmaceutical in the hepatic artery can lead to higher radiopharmaceutical concentrations and hence and improvement in treatment response, with a potential reduction in treatment toxicity (Figure 4). A high first-pass effect has already been described with the IA administration of ^68^Ga-DOTATOC compared with intravenous (IV) administration in 15 patients with GEP-NETs. IA administration of ^68^Ga-DOTATOC resulted in an average increase in SUV of 3.75-fold higher in liver metastases and 1.44- to 7.8-fold higher (dependent on the catheter placement) in the primary tumor, all compared with IV administration [150].

Kratochwil et al. performed a pilot study investigating the efficacy of hepatic IA ^90^Y-DOTATOC and/or ^177^Lu-DOTATOC in 15 patients with liver metastases from GEP-NETs [151]. An ORR of 60% and DCR of 100% was achieved. No hepatic toxicity was observed. A pharmacokinetic study with ^111^In-DOTATOC was also performed in this study. The time-activity curve of the intra-arterial administration of ^111^In-DOTATOC revealed a 3.5-fold higher uptake ratio just after termination of the infusion compared with IV administration. However, the time-activity curve of the IA administration also showed a saturation phase followed by a washout phase; this washout phase was not visible in the IV administration. This can be explained by receptor saturation, which depends on the total amount of administered peptide and the route of administration of the radiopharmaceutical (IA versus IV). Despite the washout effect, a higher tumor uptake was visible at 4 h (2-fold increase in uptake ratio) and 72 h post-injection (1.3-fold increase in uptake ratio) with the IA method, compared with the IV administration [151]. Further data are needed to further investigate the optimal peptide mass when administering PRRT intra-arterially.

## 5. Response Prediction

Another way of PRRT optimization can be achieved by pretreatment patient stratification. This strategy is gaining popularity over the last few years, given the fact that 15–30% of the patients show progression during PRRT and 10–20% of patients progress within the year after termination of PRRT [38,47,53,64,152]. Several predictors have been investigated in this setting, which include molecular or imaging markers of response. It is of utmost importance to recognize that there is a difference between prognostic factors, which can influence patient outcome, and predictors, which identify parameters associated with treatment response regardless of prognostic factors [152].

### 5.1. Molecular Testing

The PRRT predictive quotient (PPQ) allows pre-PRRT patient stratification into PRRT-responders (PPQ positive) and PRRT-non-responders (PPQ negative). PPQ is based on serum-circulating gene clusters combined with tissue-tumor grading [153]. The circulating gene clusters consist of growth factor and metabolomic genes, which have a roll in hypoxia, oxidative stress and cell metabolism. It is presumed that an increased expression of these genes translates to more radiosensitive tumors, which is the reason why a high PPQ is associated with response to PRRT [154]. PPQ revealed to be a specific predictor of efficacy in patients treated with PRRT with a high accuracy of 95%, which was determined by 3 independent European patient cohorts [154]. Further validation in a randomized setting seems warranted before widespread clinical use.

### 5.2. Imaging

Several attempts have been made to discover a predictive relationship between quantitative PET-derived parameters on SSTR-imaging and treatment response after PRRT. Öksuz et al. found that a pretherapeutic SUV_max_ of more than 17.9, derived from ^68^Ga-DOTATOC PET, can predict responders from non-responders after PRRT with ^90^Y-DOTATOC, with a sensitivity of 100% and specificity of 95% [155]. Moreover, Kratochwil et al. proposed that an SUV_max_ of more than 16.4, derived from liver metastases on ^68^Ga-DOTATOC PET, can predict treatment response after PRRT with ^90^Y/^177^Lu-DOTATOC with a sensitivity of 95% and specificity of 60% [156]. Sharma et al. found that the pretherapeutic SUV_max_ of a single lesion and the average of up to five lesions (maximum two target lesions per organ) on ^68^Ga-DOTATATE PET/CT predicted response after ^177^Lu-DOTATATE [157]. A baseline single lesion SUV_max_ cut-off of 13.0 was most optimal (sensitivity 83%, specificity 84%; *p* = 0.031). This cut-off value was also associated with longer PFS (median 45.1 months if >13.0 compared to 19.9 if <13.0) with HR 2.5 (95%CI: 1.06–6.09). Ortega et al. investigated the relationship between multiple quantitative parameters, derived from ^68^Ga-DOTATATE PET/CT at baseline and after 1 cycle of PRRT, and treatment response in 91 patients with progressive metastatic NETs [158]. A higher mean SUV_max_ of malignant lesions on baseline and interim PET and mean higher SUV_max_ tumor-to-liver ratio of malignant lesions on baseline PET were predictive of therapy response (*p* = 0.018, *p* = 0.048 and *p* = 0.024, respectively). Higher values of kurtosis, a first-order heterogeneity parameter, derived from the malignant lesions on baseline PET, were observed in non-responders compared to responders (mean 8.6 versus 5.8, respectively, *p* = 0.031). Further, Haug et al. investigated the role of ^68^Ga-DOTATATE PET/CT in early response prediction after PRRT (^90^Y/^177^Lu-DOTATATE), by evaluating changes in SUV_max_ and tumor-to-spleen SUV ratio (SUV_T/S_) between baseline and interim ^68^Ga-DOTATATE PET 3 months after the first cycle [159]. Only a decrease in SUV_T/S_ after the first cycle of PRRT was a significant predictor of time-to-progression in multivariate analysis. To the contrary, several studies did not find a predictive relationship between SUV and treatment response [160,161]. The heterogeneity in literature can be explained by the differences in used PET- and PRRT-radiopharmaceuticals, patient populations, included lesions in the analyses and PET reconstruction parameters. One important factor is the exclusion of patients with low uptake from treatment, which skews uptake/effect relationships. Large prospective studies are awaited investigating the predictive relationship of quantitative PET-derived parameters on SSTR-imaging and treatment response after PRRT. In addition to SUV_max_, the total tumor burden should also be assessed via SUV_mean_ or total lesion activity [43]. Further studies are needed to validate these findings.

## 6. Conclusions

**Figure 5:**
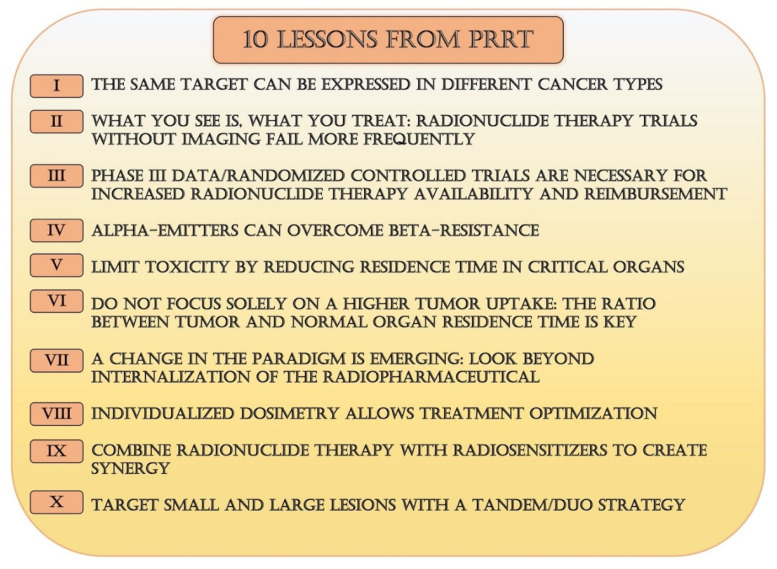
Ten lessons from PRRT. PRRT = peptide receptor radionuclide therapy.

The phase-III NETTER-1 RCT has proven that PRRT with ^177^Lu-DOTATATE results in a pronounced longer PFS and a significantly higher response rate compared to high-dose octreotide long-acting-release [70]. In addition, the NETTER-1 trial has confirmed that PRRT causes a significant improvement in the QoL of patients and aids to substantially reduce tumoral symptoms (e.g., abdominal pain, diarrhea, and flushing) [72,73]. The findings of the NETTER-1 trial combined with the results of numerous retrospective/prospective single-arm PRRT studies, established PRRT as a validated treatment for patients with advanced NETs. The evolution and continuous refinement of PRRT in the last two decades has resulted in multiple promising optimization strategies, i.e., exploiting the dose-effect concept, personalized activity administration through dosimetry, combination therapies (i.e., tandem/duo PRRT, chemoPRRT, targeted molecular therapy, somatostatin analogues and radiosensitizers), new radiopharmaceuticals (i.e., SSTR-antagonists, vector molecules with increased plasma half-life and alpha-emitters), administration route (intra-arterial versus intravenous) and response prediction via molecular testing or imaging. The results of prospective trials exploring these optimization strategies are strongly awaited and can hopefully lead to a further increase in treatment efficacy of PRRT. Finally, the lessons learnt from the development of PRRT will accelerate the future development of RNT involving other targets, vectors and/or radionuclides (Figure 5).

## Figures and Tables

**Table 1:** SSTR affinity profiles for peptide receptor radionuclide therapy.

	SSTR Affinity				
Somatostatin Analogue	SSTR 1	SSTR 2	SSTR 3	SSTR 4	SSTR 5
^111^In-DTPA-octreotide [5]	>10,000	22 ± 3.6	182 ± 13	>1000	237 ± 52
^90^Y-DOTATOC [5]	>10,000	11 ± 1.7	389 ± 135	>10,000	114 ± 29
^90^Y-DOTATATE [5]	>10,000	1.6 ± 0.4	>1000	523 ± 239	187 ± 50
^177^Lu-DOTATATE [6]	>1000	2.0 ± 0.8	162 ± 16	>1000	>1000
^177^Lu-DOTA-JR11 [7]	>1000	0.73 ± 0.15	>1000	>1000	>1000

All values are IC_50_ ± standard error of the mean, in nM. SSTR = somatostatin receptor.

**Table 2:** Efficacy and outcome in PRRT.

First Author	Design	*n*	Subtype	Setting	Compound	ORR	DCR	CR	PR	MR	SD	PD	Criteria	Median PFS (mo)	Median OS (mo)	Comments
Cohorts																
Imhof [23]	P	1109	GEP-lung-other-CUP NET/neural crest	Disease progression	^90^Y-DOTATOC	34%	39%	1%	34%	-	5%	61%	Simplified response criteria ^1^	NA	NA	
Brabander [45]	R	443	GEP-lung-other-CUP NET	Imaging progression/clinical progression/high tumor load	^177^Lu-DOTATATE	39%	83%	2%	37%	-	43%	12%	RECIST 1.1	29	63	
Hörsch [46]	R + P	450	EP-lung-CUP-NEN	Progression/locally advanced disease/metastatic disease	^90^Y/^177^Lu-DOTATOC/ DOTATATE	35%	95%	7%	28%	-	59%	5%	RECIST 1.1	41	59	
Kwekkeboom [47]	P	310	GEP-NET	Imaging progression 43%/Other	^177^Lu-DOTATATE	46%	80%	2%	28%	16%	35%	20%	SWOG	33	46	
Garske-Roman [48]	P	200	GEP-lung-CUP-other NET/NEC/neural crest	Progression (81%) > first line treatment metastatic rectal NETs or bronchopulmonary carcinoids (19%)	^177^Lu-DOTATATE	24%	92%	1%	24%	-	68%	4%	RECIST 1.1	27	43	
Hamiditabar [49]	P	143	GEP-lung-other-CUP NET/neural crest	Imaging progression	^177^Lu-DOTATATE	8%	55%	0%	8%	-	46%	38%	RECIST	NR	NR	
Mariniello [50]	R	114	lung-NET	Imaging progression (78%) > other	^90^Y-DOTATOC/ ^177^Lu-DOTATATE/^90^Y-DOTATOC + ^177^Lu-DOTATATE	27%	67%	0%	13%	13%	41%	33%	RECIST	28	59	
Kunikowska [51]	R	103	EP-lung-CUP-other NET/neural crest	Metastatic, inoperable disease	^90^Y-DOTATATE ^177^Lu-DOTATATE	24%	88%	2%	22%	-	64%	12%	RECIST 1.1 + SRS	30	90	Tandem PRRT
Fröss-Baron [52]	R	102	pNET	Imaging progression (90%) > intolerance to previous therapies (8%) > pseudo-neoadjuvant (2%)	^177^Lu-DOTATATE	49%	91%	4%	45%	-	44%	7%	RECIST 1.1	24	42	
Ezziddin [53]	R	74	GEP-NET	Imaging progression (76%) > clinical progression (22%) > uncontrolled disease under SSA (11%)	^177^Lu-DOTATATE	37%	89%	0%	37%	18%	35%	11%	mSWOG	26	55	
Pfeifer [54]	R	69	GEP-lung-CUP NET	Imaging progression (81%) > intolerance previous therapies (19%)	^90^Y-DOTATOC ^177^Lu-DOTATOC	24%	56%	7%	16%	-	62%	15%	RECIST	29	NR	
Campana [55]	R of P database	69	GEP-NET	Imaging progression (51%)/advanced disease not suitable for radical surgery/residual disease after debulking surgery	^90^Y-DOTATOC ^177^Lu-DOTATATE	28%	78%	0%	28%	-	51%	23%	RECIST	28	NA	
Ezziddin [56]	R	68	pNET	Imaging progression (68%) > high tumor burden (19%) > clinical progression (13%)	^177^Lu-DOTATATE	60%	85%	0%	60%	12%	13%	15%	mSWOG	34	53	
Sabet [57]	R	61	SI-NET	Imaging progression (75%) > clinical progression (25%)	^177^Lu-DOTATATE	13%	92%	0%	13%	31%	48%	8%	mSWOG	33	61	
Sansovini [58]	P	60	pNET	Imaging progression/unresectable or metastatic disease	^177^Lu-DOTATATE	30%	82%	7%	23%	-	52%	18%	SWOG	29	NR	
Kunikowska [59]	P	59	EP-lung-CUP-other NET/neural crest	Clinical progression/imaging progression/ biochemical progression	^90^Y-DOTATATE ^177^Lu-DOTATATE	24%	89%	2%	22%	-	65%	6%	RECIST 1.1	32	82	Tandem PRRT
Vinjamuri [60]	R	57	GEP-lung-other-CUP NET	Clinical progression (45%) > imaging progression (33%) > clinical and imaging progression (22%)	^90^Y-DOTATOC ^90^Y-DOTATATE	25%	72%	0%	25%	-	47%	29%	RECIST	NA	46	
Baum [61]	R	56	GEP-lung-CUP-other NET	Imaging progression	^177^Lu-DOTATOC	34%	66%	16%	18%	-	32%	34%	RECIST 1.1	17	34	
Del Prete [62]	P	52	GEP-lung-CUP-NET/neural crest	Progressive and/or symptomatic NET	^177^Lu-DOTATATE	36%	82%	0%	18%	18%	46%	18%	RECIST 1.1	16	NR	Only 11 patients were available for response assessment
Bodei [63]	P	51	EP-lung-CUP NET/neural crest	Imaging progression > other	^177^Lu-DOTATATE	29%	82%	2%	27%	26%	27%	18%	RECIST	median TTP = 36 mo	NR	
Zidan and Iravani [64]	R	48	lung-NET	Imaging progression (98%) > uncontrolled symptoms (2%)	^177^Lu-DOTATATE	20%	88%	0%	20%	-	68%	12%	RECIST 1.1	23	59	33% patients received chemo-PRRT
Paganelli [65,66]	P	43	GE-NET	Imaging progression	^177^Lu-DOTATATE	7%	84%	0%	7%	-	77%	16%	SWOG	60	82	
Pauwels [43]	R of P trial	43	GEP-CUP-other NET	Clinical/imaging progression	^90^Y-DOTATOC	0%	55%	0%	0%	-	55%	45%	RECIST 1.1	14	22	
Ianniello [67]	P	34	lung-NET	Imaging progression	^177^Lu-DOTATATE	15%	62%	3%	12%	-	47%	38%	SWOG	19	49	
Zandee [68]	R	34	functioning pNET	Imaging progression (41%) > symptom reduction (27%) > imaging progression and symptom reduction (24%) > high tumor burden (9%)	^177^Lu-DOTATATE	59%	78%	3%	56%	-	24%	18%	RECIST 1.1	18	NA	71% reduction of syndrome-specific symptoms after PRRT
Zandee [69]	R	30	neural crest	Symptomatology/imaging progression/high tumor burden.	^177^Lu-DOTATATE	23%	85%	0%	23%	-	68%	10%	RECIST 1.1	30	NR	
**RCT**																
Strosberg [70,71]	RCT	116	midgut-NET	Imaging progression	^177^Lu-DOTATATE	18%	NA	1%	17%	-	NA	NA	RECIST 1.1	NR	48	

*n* = number of included patients, ORR = objective response rate, DCR = disease control rate, CR = complete response, PR = partial response, MR = minor response, SD = stable disease, PD = progressive disease, PFS = progression-free survival, OS = overall survival, NET = neuroendocrine tumor, GEP = gastroenteropancreatic, CUP = unknown primary tumor, pNET = pancreatic NET, EP = enteropancreatic, SI-NET = small intestine NET, NEC = neuroendocrine carcinoma, NEN = neuroendocrine neoplasm, RECIST = Response Evaluation Criteria in Solid Tumors, SWOG = Southwest Oncology Group, mSWOG = modified SWOG, SRS = somatostatin receptor scintigraphy, NA = not available, NR = not reported, PRRT = peptide receptor radionuclide therapy, RCT = randomized controlled trial, P = prospective, R = retrospective, SSA = somatostatin analogue, mo = months, TTP = time to progression. ^1^ Response was defined as any measurable decrease in the sum of the longest diameters of all pretherapeutically detected tumor lesions. CR was defined as disappearance of all lesions, MR as concurrence of increasing and decreasing lesions, SD if no changes occurred and PD was defined as any measurable increase in the sum of the longest diameters of the pretherapeutically detected lesions.
